# Single camera multi-view anthropometric measurement of human height and mid-upper arm circumference using linear regression

**DOI:** 10.1371/journal.pone.0195600

**Published:** 2018-04-12

**Authors:** Yingying Liu, Arcot Sowmya, Heba Khamis

**Affiliations:** 1 Graduate School of Biomedical Engineering, University of New South Wales, Sydney, Australia; 2 School of Computer Science and Engineering, University of New South Wales, Sydney, Australia; TNO, NETHERLANDS

## Abstract

**Background:**

Manually measured anthropometric quantities are used in many applications including human malnutrition assessment. Training is required to collect anthropometric measurements manually, which is not ideal in resource-constrained environments. Photogrammetric methods have been gaining attention in recent years, due to the availability and affordability of digital cameras.

**Objective:**

The primary goal is to demonstrate that height and mid-upper arm circumference (MUAC)–indicators of malnutrition–can be accurately estimated by applying linear regression to distance measurements from photographs of participants taken from five views, and determine the optimal view combinations. A secondary goal is to observe the effect on estimate error of two approaches which reduce complexity of the setup, computational requirements and the expertise required of the observer.

**Methods:**

Thirty-one participants (11 female, 20 male; 18–37 years) were photographed from five views. Distances were computed using both camera calibration and reference object techniques from manually annotated photos. To estimate height, linear regression was applied to the distances between the top of the participants head and the floor, as well as the height of a bounding box enclosing the participant’s silhouette which eliminates the need to identify the floor. To estimate MUAC, linear regression was applied to the mid-upper arm width. Estimates were computed for all view combinations and performance was compared to other photogrammetric methods from the literature—linear distance method for height, and shape models for MUAC.

**Results:**

The mean absolute difference (MAD) between the linear regression estimates and manual measurements were smaller compared to other methods. For the optimal view combinations (smallest MAD), the technical error of measurement and coefficient of reliability also indicate the linear regression methods are more reliable. The optimal view combination was the front and side views. When estimating height by linear regression of the distance from the head to the floor, the mean MAD was 10.51 mm ± 6.52 mm SD, and when estimating height from the bounding box using the reference object, the mean MAD per participant was 11.53 mm ± 6.43 mm SD. When estimating MUAC from the mid-upper arm radius using the reference object, the mean MAD was 7.24 mm ± 4.79 mm SD. The mean MAD for all methods when using camera calibration was 2–3 mm smaller.

**Conclusions:**

Applying linear regression to distance measurements from photos of adults taken from multiple view angles has been shown to accurately estimate height and MUAC to within the accuracy required for nutrition assessment. Future work will focus on automating the landmark detection, and validating the methods on populations that include undernourished adults and children of all nutrition statuses. These future works will improve the practicality of this method as a potential tool for nutrition assessment by novice users.

## Introduction

Anthropometric quantities describe the size, shape and composition of the human body. Such quantities have many applications in product design [1] and clothing sizing [2], and are also important for assessing health and nutrition status in children and adults. [3–5]. Two commonly used anthropometric quantities for assessing malnutrition and obesity in children, and undernutrition in adults, are height and mid-upper arm circumference [6–9]. In anthropometric studies, height and MUAC are typically measured manually, multiple times, by one or more trained observers, making the measurements time-consuming, inconvenient and also impractical in resource-constrained environments. There is also concern regarding the reliability of manual anthropometric measurements, due to observed inter- and intra- operator measurement variability [10,11]. As digital cameras have become more affordable and with increasing penetration of smart-phones with on-board digital cameras in emerging markets [12,13], digital cameras are being widely used in various fields, including anthropometric measurement.

In photogrammetric anthropometry, height is often estimated as the linear distance between two landmarks on the participant's head and feet. The top point of the participant's head and the center point between the participant's feet have been used previously with the participant standing with their heels, buttocks and head against a wall [14], however, the center point between the participant’s feet requires a physical marker (usually drawn on the floor between the participant’s feet), as identifying this point without a marker is ambiguous. Moreover, for multiple views, the center point is not always visible (e.g. side view), and for this reason, typically only photos from the participant's frontal view are used for height estimation. Frame by frame video analysis has also been used to measure height [15].

Photogrammetric methods have also been applied to estimate various circumferential measurements. Shape models including circle, ellipse and rectangle have previously been used for circumferential measurements, for example forehead, neck, chest, waist, and hip circumferences, which were approximated as the perimeters of the shape models estimated from two photos taken from the frontal and side views of the participant [2,16,17]. Among these models, the ellipse model reportedly produced the smallest mean absolute difference (MAD) against manual measurements. Besides shape models, some researchers estimate waist and hip circumferences by fitting a surface to a 3D model of the human body which is reconstructed from multiple photos using cubic splines [18,19].

In this study, linear regression is applied to distance measurements from photographs of adult participants taken from multiple views to estimate height and MUAC. Linear regression has previously been applied to estimate the height based on length in the frontal view, and circumferences of the arm, thigh and calf based on their widths from frontal and lateral views [20]. However the estimate errors were not reported; instead, the output of the regression was used as an input to neural network models to estimate the Z-score values of weight-for-age, height-for-age, and weight-for-height, and the Z-score values were then used to classify the child’s malnutrition status. The primary goal in this work is to demonstrate that height and MUAC can be accurately estimated in this way, as well as determine the optimal view combinations (giving the smallest measurement error). A secondary goal is to observe the effect on estimation error of attempting to reduce the complexity of the setup and the expertise required of the observer (who is taking the photographs). Both camera calibration and reference object methods are used to compute distances in real world units, to compare the measurement error from both techniques. For height, in addition to the distance between the top of the participant's head and the floor, the height of a bounding box of the participant’s entire silhouette was also used. For MUAC, the width of the mid-upper arms was computed from photos. Linear regression was applied to the distance measurements for height and mid-upper arm width from all combinations of five views. Comparisons were conducted between manual measurements, the proposed linear regression methods and previously used methods, i.e., linear distance for height estimation and shape models for MUAC estimation. Furthermore in this study, shape models are exhaustively evaluated for MUAC estimation using photos from five views, instead of only two views (frontal and side views) as in the published literature [16].

## Methods

### Dataset

A dataset consisting of manually-measured heights and left and right MUAC as well as photos of 31 participants (11 female, 20 male; mean age 26.2 years ± 4.8 SD), was created for evaluation. Data collection was performed with approval from the Human Research Ethics Committee B at the UNSW Sydney (approval number: HC16907) and all participants provided informed written consent. Participants under 18 years of age and people with low mobility were excluded from the study. Fifty potential participants were approached verbally between October 2016 and April 2017, and data collection from 31 consenting participants was performed during the same period. No participants dropped out of the study. Manual measurement and photo capture of every participant were performed in the same session and completed within an hour avoiding the effects of daily body variations.

#### Manual measurement

Manual measurements were collected by the researcher according to the WHO anthropometry training course [21] and as described in [22].

Briefly, the height was measured with the subject standing with feet slightly apart with the back of their head, their buttocks and their heels against a wall with the head positioned so that the horizontal line connecting the upper ear opening and the lower edge of the eye socket runs parallel to the floor; a laser range finder (Bosch GLM 7000; Class II laser, accuracy ± 1.5 mm) was used to measure the distance from the top of the subjects head to the ceiling, which was subtracted from the distance between the floor and the ceiling (measured with the same device). Participants were free to remove or remain in their footwear during height measurement for comfort and convenience. While this point is not in line with the WHO guidelines, which states that shoes and socks are to be removed, during photo capture participants footwear remained as it was during manual measurement, therefore the photogrammetric height estimate corresponds to the manual height measurement and this deviation from the WHO guidelines does not detract from the meaningfulness of the work.

The mid-upper arm is the midpoint between the acromion process (bony process on the shoulder blade) and the elbow (when the elbow is bent at approximately a 90° angle). Following identification of the mid-upper arm, the subject then relaxed their arm by their side and an un-stretchable tape measure (Ibis Medical; Infant head circumference measuring tape) was wrapped around the arm passing over the midpoint, touching the skin without compressing the tissues and skin; the MUAC is recorded to the nearest mm. In order to indicate the mid-upper arm in photographs, lines were drawn on both arms of the participants when their manual measurements were collected.

Each anthropometric quantity was measured three times by a single observer (the researcher) for each participant, and the average measurement was taken as the ground truth. The age, weight, manually-measured height and left and right MUAC of the participants are summarized in Table 1.

**Table 1:** A summary of the age, body mass index (BMI), weight, height and left and right mid upper arm circumference (MUAC) of the 31 study participants (11 female, 20 male).

	Mean	SD	Min	Max
Age (years)	26.2	4.8	18.0	37.0
BMI (kg/m^2^)	22.8	3.2	18.0	30.6
Weight (kg)	66.7	12.6	42.0	93.0
Height (mm)	1706.5	89.1	1526.3	1843.7
Left MUAC (mm)	291.2	39.1	225.3	364.0
Right MUAC (mm)	294.1	37.5	230.7	373.3

#### Photo capture

Photos of the participants were collected indoors with a tripod mounted Canon IXUS 110 IS digital camera with a resolution of 4000×3000 pixels. The camera was set to auto focus, auto exposure time and ISO-200, and no zoom or image stabilization was applied. Participants’ arms were bare from the shoulder down, and participants remained barefoot or in their footwear (as they were during the manual measurement of height) for their comfort and convenience.

In some previous photogrammetry literature, where only the front view is used, the participant is standing with their heels, buttocks and head against a back wall [14] (as is required during the manual measurement of height). In the case of multi-view photogrammetry, it is preferable that the participant maintains the same pose/posture in photographs from all views, and since standing against a wall is not possible in all views (consider when the participant’s back is towards the camera), the participant is required to be free-standing. Similarly, in some previous literature, the participant is instructed to stand in the anatomical position–i.e. with the arms making a 45° angle with the median line of the body and palms facing forwards [14]. This is a different pose than that used for manual measurement of MUAC (arms by the side), and the activation of muscles in the upper arm may affect the photogrammetric measurement of MUAC, and it is therefore preferable that the participant remain with their arms by their side.

In this study, during photo capture, participants were instructed to stand up straight, and look straight ahead with their arms by their side during photo capture with their medial line in the same plane as a checkerboard (with 7×10 squares, each square of which is 90×90 mm) which was perpendicular to the floor next to the participant.

**Fig 1:**
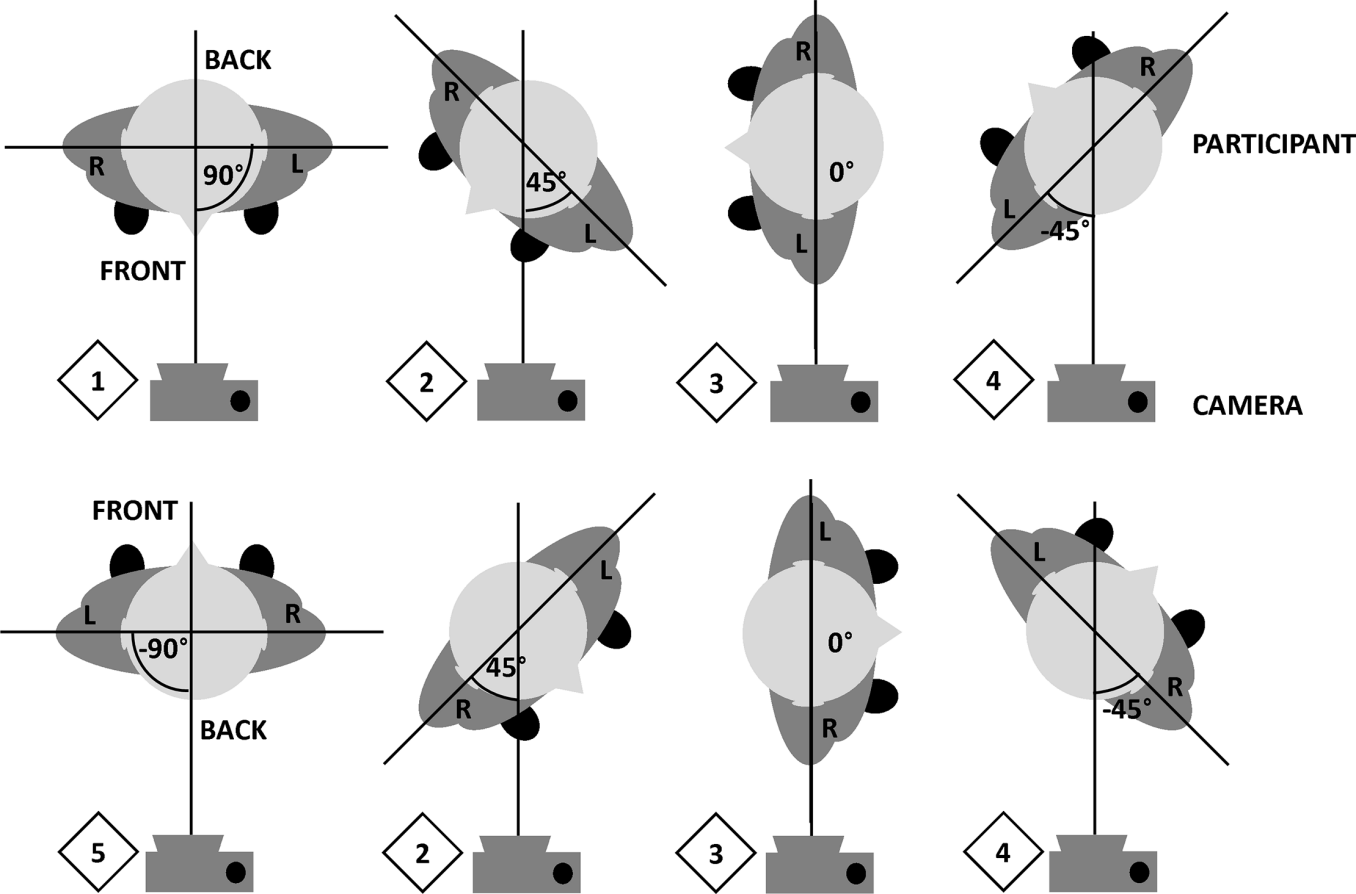
Illustration of photo capture setup: Views 1 to 5 –relative orientations between the camera and the participant’s left and right arm.

Photos of each participant were taken from five views by changing the orientation of the participant relative to the camera lens—for the different views, the camera and checkerboard positions were fixed and the subject would turn and reposition their feet according to markings on the ground, whilst keeping their medial line in the same plane as the checkerboard. For different relative angles between the camera and the participant, the left or right arm may be occluded by the torso or clothing. Here, five views are defined with respect to the arm that is not obstructed. The views are labeled according to the relative angle made between the line connecting the camera and the medial line of the participant, and the line connecting the closest shoulder and the furthest shoulder as shown in Fig 1. View 1 and view 5 are the frontal and back views of the participant respectively, with both arms unobstructed in each; in view 2 the arm is at a 45° angle with the line connecting the camera and the medial line of the participant passing through the front of the body (two different photos with only one arm unobstructed in each); view 3 refers to the side view (two different photos with only one arm visible in each); and finally in view 4 the arm is at a 45° angle with the line connecting the camera and the medial line of the participant passing through the back of the body (two different photos with only one arm obstructed in each).

A total of 744 photos were taken at three distances between the participants and the camera lens (i.e. three distances per participant: mean 3.39 m ± 0.30 m SD, mean 3.25 m ± 0.33 m SD and 3.12 m ± 0.32 m SD, respectively, with the difference between the furthest and the middle distance, and between the middle and the closest distance approximately equal to 150 mm). The camera was approximately 1.125 m above the floor. Variation in the camera-participant distances and the height of the camera were due to the equipment being set-up/packed-up in different locations (rooms of varying sizes) before/after every participant.

#### Manual labels

Each photo was manually annotated with a number of markers:

■ Reference object: The paper on which the calibration checkerboard was also used as a reference object within the image. The corners of the paper were manually marked. The dimensions of the paper were 840 × 1186 mm.
■ Floor line: The two corners at the bottom of the calibration checkerboard were marked as an indication of the location of the floor in the plane of the checkerboard (and hence the participant).
■ Top of the head: the top of the participants head was marked
■ Bounding box: The bounding box enclosing the silhouette of the participant was marked with the top edge passing through the top of the head, and the bottom edge passing through the lowest point of the feet.
■ Edges of the left and right mid-upper arm: The edges of the left and right mid-upper arm were marked where the MUAC had been measured manually (indicated by a line drawn on the skin).

**Fig 2:**
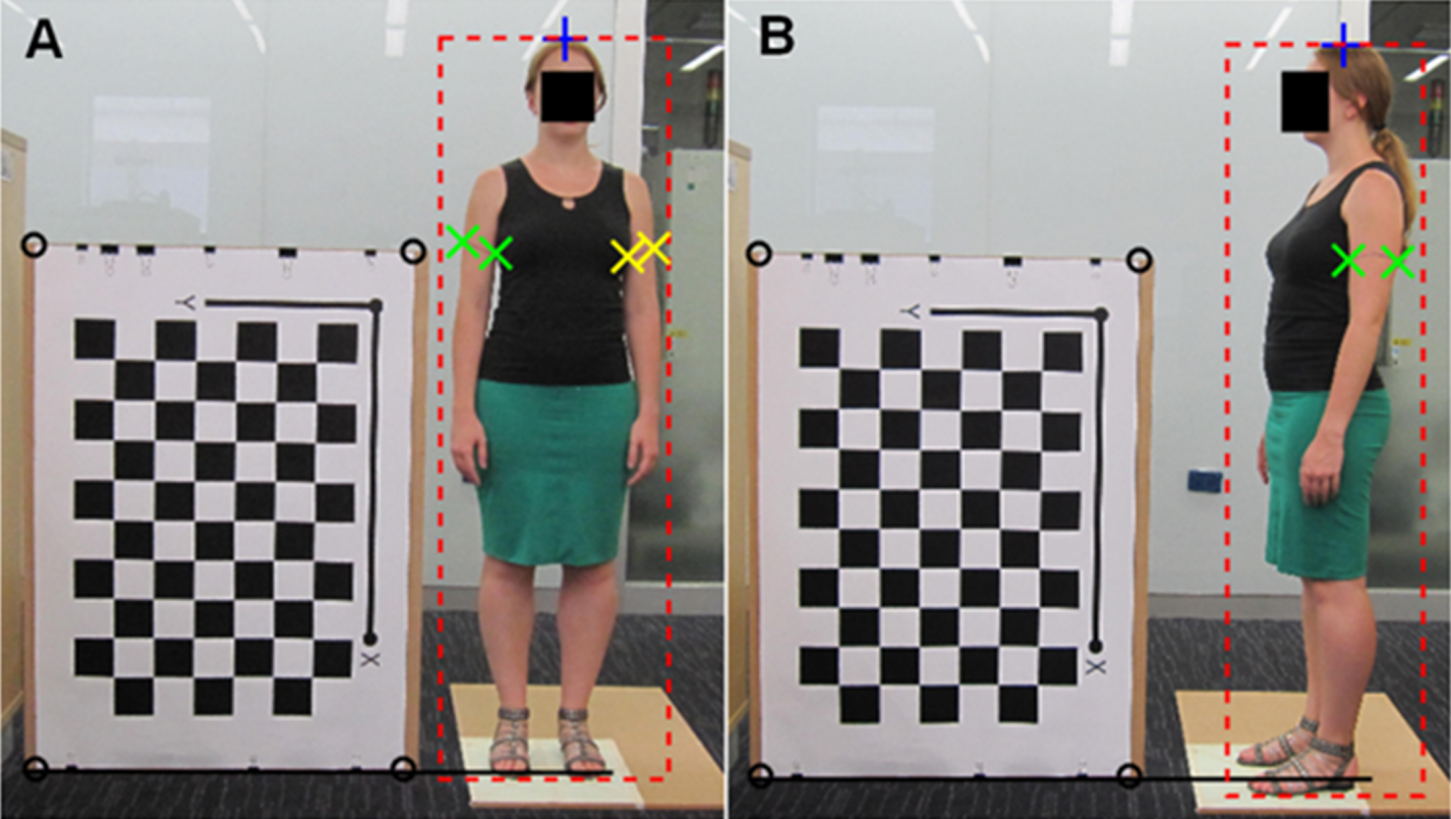
Manually annotated markers of a photo from A) view 1, and B) view 3, of participant 0007. Black circles are the corners of the reference object; black solid line is the floor line; blue cross is the top of the head; red dashed line is the bounding box; green x’s are the two edges of the left mid-upper arm; and yellow x’s are the two edges of the right mid-upper arm. Note that the floor line and the bottom edge of the bounding box do not align.

An example of the manually annotated markers for two photos (from view 1 and view 3) is shown in Fig 2.

### Calculating distance in an image

Two methods for calculating the real world distance between two points in the image were applied here: i) using camera calibration, and ii) using the reference object. While in general, a reference object method is less accurate than a calibrated camera method, the reference object method is simpler, in terms of photo capture complexity, and the expertise required of the observer, and furthermore, the reference object method requires less computational power than the camera calibration method.

#### Camera calibration

Camera calibration is often used to back-project the two-dimensional image coordinates to their three-dimensional world coordinates for measurement estimation. The camera intrinsic and extrinsic parameters, such as translation and rotation matrices, as well as distortion coefficients, were determined using the MATLAB (Release 2016a, The MathWorks, Inc., Natick, USA) Single Camera Calibration App [23]. Fifteen photos were taken of the checkerboard (with 7×10 squares, each square of which is 90×90 mm) in various positions and orientations. The Calibration App automatically detects the corners of the squares in each of the calibration photos and applies a calibration algorithm which assumes a pinhole camera model to determine the camera parameters. The camera calibration process was performed just once and the calculated camera parameters were then used to undistort all photos of participants prior to any further processing. The camera parameters were also used for transforming image coordinates (in pixels) to world coordinates (in mm). Distances are then calculated in real world units (mm) directly from the world coordinates.

#### Reference object

Below is a description of the computation of a distance in real world units using the reference object:

1. The vertical distance and horizontal distance between two points in the original photo (i.e. without being undistorted using the camera parameters) is calculated in image coordinates (pixels). To convert a vertical distance in pixels to real world units, the height of the reference object in both pixels and mm is needed. Similarly, to convert a horizontal distance in pixels to real world units, the width of the reference object in both pixels and mm is needed.
2. The reference object height in pixels is determined at the horizontal position at which the vertical distance is being measured. The line connecting the top left and top right corners of the reference object and the line connecting the bottom left and bottom right corners of the reference object are both extrapolated. The distance between these two lines at their intersection with the vertical line where the vertical distance is being measured, is taken to be the reference object height in pixels (*h*_*pi*_). The reference object height in real world units (*h*_*mm*_) is 1186 mm.
3. The reference object width in pixels is determined at the height at which the horizontal distance is being measured by extrapolating the line connecting the top left and bottom left corners of the reference object and extrapolating the line connecting the top right and bottom right corners of the reference object. The distance between these two lines at their intersection with the horizontal line where the horizontal distance is being measured, is taken to be the reference object width in pixels *w*_*pi*_. The reference object width in real world units *w*_*mm*_ is 840 mm.
4. The vertical distance in pixels is converted to real world units (mm) by multiplying by the ratio *h*_*mm*_/*h*_*pi*_. The horizontal distance in pixels is converted to real world units (mm) by multiplying by the ratio *w*_*mm*_/*w*_*pi*_. Finally, the Euclidian distance is taken as the square root of the sum of squares of the vertical and horizontal distances in real world units.

### Height estimation

Height is often estimated as the distance between the top of the head and a marker on the floor at the center of the participant’s feet [14]. In multi-view photogrammetry, such a marker can be occluded by one of the participants feet (consider the side-view–view 3 –where the foot closest to the camera occludes the floor between the participant’s feet as in Fig 2B), and therefore an alternative method to identifying the location of the floor is required. Here, the floor is identified by extrapolating the line connecting the corners of the bottom edge of the reference object/checkerboard which is in contact with the floor (see Fig 2). This necessitates that the reference object/checkerboard, must have a straight edge which must be positioned on the floor. An approach that is less restrictive since it does not require the reference object/checkerboard to be positioned on the floor, and indeed does not require that the floor be identified at all, is also considered here, which uses a bounding box that encloses the entire silhouette of the participant (from the top of the head to the lowest point of the feet–see Fig 2). The bottom edge of the bounding box is not indicative of the location of the floor at the center of the feet, since the lowest point of the feet may be closer to the camera, and therefore it can appear lower than the floor line in the photograph–see Fig 2B.

Here, three methods were applied to estimate height: (i) a linear distance method that approximates height as the Euclidean distance between the top of the head and the floor line, (ii) linear regression of the Euclidean distance between the top of the head and the floor line, and (iii) linear regression of the mean of the height of the bounding box.

#### Linear distance

The closest point on the floor line (*ax* + *by* + *c* = 0) to the head point *P*_*H*_ = (*x*_0_,*y*_0_) is the point *P*_*H*_ = (*x*_1_,*y*_1_):

$$x_{1}=\frac{b\left(bx_{0}-ay_{0}\right)-ac}{a^{2}+b^{2}},\;y_{1}=\frac{a\left(-bx_{0}+ay_{0}\right)-bc}{a^{2}+b^{2}}$$ (1)

The linear distance estimate of the height of the participant is:

$$H_{LD}=\left|P_{H}-P_{F}\right|.$$ (2)

In the case of using camera calibration, the points *P*_*H*_ and *P*_*F*_ are converted to world coordinates before applying Eq (2). In the case of using the reference object, the Euclidian distance between *P*_*H*_ and *P*_*F*_ is first calculated in pixels and then this distance is converted into mm (as described in the section “Calculating distances in an image–Reference object”) to give *H*_*LD*_.

#### Regression of linear distance

A linear regression model was applied to the linear distance height estimate calculated from one or more views:

$$H_{LDReg}=\sum_{i\in S}\left(w_{i}H_{LD}^{i}\right)+b$$ (3)

where $H_{LD}^{i}$ is the *H*_*LD*_ measurement (as above) from a photo taken from view *i* using the linear distance method, *w*_*i*_ is its weight, *b* is a bias coefficient, and *S* is a non-empty subset of {1, 2, 3, 4, 5}, which contains between one and five numbers that represent the views that are involved in the estimate.

#### Regression of bounding box height

The four corners of the participant bounding box are A: top left, B: top right, C: bottom left, and D: bottom right. The height of the bounding box is then taken as:

$$H_{BB}=\left(d_{AC}+d_{BD}\right)/2$$ (4)

where *d*_*AC*_ is the Euclidean distance between *A* and *C* and *d*_*BD*_ is the Euclidean distance between *B* and *D*.

In the case of camera calibration, the points *A*, *B*, *C* and *D* are the world coordinates of the corners of the bounding box. *H*_*BB*_ is roughly equivalent to the distance between the midpoint of the top edge and the midpoint of the bottom edge of the bounding box, which, assuming the participant is in the center of the bounding box, correspond approximately to the center of the top of the head and the center of the bottom of the feet, respectively. In the case of using a reference object, the points *A*, *B*, *C* and *D* are the image coordinates of the corners of the bounding box, and the distances *d*_*AC*_ and *d*_*BD*_ are therefore in pixels (and, in fact, *d*_*AC*_ = *d*_*BD*_). These are then converted to world distances in mm (as described in the section “Calculating distances in an image–Reference object”), before applying Eq (4) to give *H*_*BB*_.

It is expected that the area of the participants' feet in a photo introduces an error to the height estimate *H*_*BB*_, since the exact location of the floor is unknown. Thus a linear regression model was applied:

$$H_{BBReg}=\sum_{i\in S}\left(w_{i}H_{BB}^{i}\right)+b$$ (5)

where $H_{BB}^{i}$ is the *H*_*BB*_ measurement (as above) from a photo taken from view *i* using the linear distance method, *w*_*i*_ is its weight, *b* is a bias coefficient, and *S* is a non-empty subset of {1, 2, 3, 4, 5}, which contains between one and five numbers that represent the views that are involved in the estimate.

#### Cross-validation

Leave-one-out cross-validation (LOOCV) was used to train and test independent linear regression models for each combination of views for the height estimate using linear regression of linear distance, and linear regression of the bounding box height. For each iteration of LOOCV, all data from a single participant was left out to learn the regression weights, and those learned weights were then used to estimate the measurement for that participant.

### MUAC estimation

The edges of the upper arms at the mid-upper arm mark (drawn during manual measurement) were labeled manually in each photo–see Fig 2. The widths of the mid-upper arms can be approximated as the Euclidean distance between the markers indicating the two edges of mid-upper arms. In the case of camera calibration, the marker points are first converted to world coordinates and then the Euclidian distance is calculated; and in the case of using the reference object, the Euclidian distance is calculated in pixels and then the distance is converted to mm (as described in the section “Calculating distances in an image–Reference object”). The radius of the mid-upper arm is taken to be half of its width.

In this study, MUAC was estimated using shape models, which have been previously used in the literature for other circumferential measurements, and also using linear regression applied to the mi-upper arm radius from combinations of views.

#### Shape models

Several shape models have been used to estimate body circumferences in previous studies [16,17]. A transverse cross-section of the body part is modeled as a particular shape, and the circumference of the cross-section is then estimated as the perimeter of that shape. Three shapes were applied to the MUAC estimation here: circle, ellipse, and a combination of ellipse and rectangle.

The circle model was applied to each photo where the participant's mid-upper arm is visible. The MUAC is estimated as:

$$M_{circ}=2\pi r_{i}$$ (6)

where *r*_*i*_ is the radius of the mid-upper arm estimated from a photo taken from view *i*.

The ellipse model was applied to pairs of photos in which the same (left or right) mid-upper arm is visible. The MUAC is approximated as:

$$M_{ell}=\pi \;\left(\left(3r_{i}+3r_{j}\right)-\sqrt{\left(r_{i}+3r_{j}\right)\left(3r_{i}+r_{j}\right)}\right)$$ (7)

where *r*_*i*_ and *r*_*j*_ refer to the radii of the mid-upper arm estimated from the two photos taken from view *i* and *j* respectively.

Similarly, the combination of ellipse and rectangle was applied to pairs of photos. The MUAC is approximated as:

$$M_{mix}=\left(2r_{i}+2r_{j}\right)+M_{ell}/2$$ (8)

where *r*_*i*_ and *r*_*j*_ refer to the radii of the mid-upper arm estimated from the two photos taken from view *i* and *j* respectively.

In previous studies [16,17], the shape models were applied to photos taken only from the frontal and side views of the participants, which correspond to view 1 and view 3, respectively, in this study (see Fig 1). To exhaustively evaluate existing shape models on MUAC estimation, the shape models were applied to photos and photo pairs from all available views in the dataset. The circle model was applied to photos from views 1, 2, 3, 4 and 5, and both the ellipse model and the combined ellipse and rectangle model were applied to photo pairs from all possible combinations of two views: {1, 2}, {1, 3}, {1, 4}, {1, 5}, {2, 3}, {2, 4}, {2, 5}, {3, 4}, {3, 5} and {4, 5}.

#### Regression of mid-upper arm radius

A linear regression model was applied to estimate MUAC from the radius of the mid-upper arm:

$$M_{reg}=\sum_{i\in S}\left(w_{i}r_{i}\right)+b$$ (9)

where *r*_*i*_ is the radius of the participant's mid-upper arm estimated from a photo taken from view *i*, *w*_*i*_ is the regression weight of the measurement from view *i*, and *b* is the bias coefficient. *S* is a non-empty subset of {1, 2, 3, 4, 5}, which refers to the views used in the estimate.

#### Cross-validation

LOOCV was used to train and test independent linear regression models for MUAC estimation for each combination of views, with the data from one arm of a single participant being left out at each iteration to learn the regression weights, and those learned weights were then used to estimate the arm circumference measurement for that participant.

### Evaluation metrics

In anthropometric studies, observers' measurements are commonly evaluated by comparing with an expert's measurements. In this study, in order to evaluate the photogrammetric methods, their estimates were compared with manual measurements. MAD, mean absolute percentage difference (MAPD) and the technical error of measurement (TEM) and coefficient of reliability (R) were computed to evaluate the performance of all the estimation methods.

#### MAD

MAD is the mean absolute difference between the photo-based estimates and the expert's measurements. The mean of manual measurements for each participant in the case of height, and for each arm of each participant, in the case of MUAC, was computed as the expert's measurement. MAD was computed for each participant. The MAD of the *i*^th^ participant is defined as:

$$MAD_{i}=\frac{\sum_{j=1}^{N_{i}}\left|x_{i}-{\hat{x}}_{ij}\right|}{N_{i}}$$ (10)

where *x*_*i*_ is the mean manual measurement for participant *i*, ${\hat{x}}_{ij}$ is the *j*^th^ estimate for participant *i*, and *N*_*i*_ is the number of estimates for participant *i*. The mean and standard deviation of *MAD*_*i*_ for all participants is computed as one measure of performance for each photo-based method.

#### MAPD

MAPD is the mean absolute percentage difference between the photo-based estimates and the expert's measurements. MAPD for each participant is also calculated. The MAPD of the *i*^th^ participant is defined as:

$$MAPD_{i}=100\%\times \frac{\sum_{j=1}^{N_{i}}\left|x_{i}-{\hat{x}}_{ij}\right|/x_{i}}{N_{i}}$$ (11)

where *x*_*i*_ is the mean manual measurement for participant *i*, ${\hat{x}}_{ij}$ is the *j*^th^ estimate for participant *i*, and *N*_*i*_ is the number of estimates for participant *i*. The mean and standard deviation of *MAPD*_*i*_ for all participants is computed as another measure of performance for each photo-based method, which is reported for completeness.

#### TEM and R

TEM and R are commonly used to evaluate the reliability of manual anthropometric measurements [24,25], and have also been used to evaluate photogrammetric anthropometric estimates in the literature [2,16]. These are defined as:

$$TEM=\sqrt{\sum_{i=1}^{M}\frac{\sum_{j}^{N_{i}}{\hat{x}}_{ij}^{2}-\frac{1}{N_{i}}{\left(\sum_{j}^{N_{i}}{\hat{x}}_{ij}\right)}^{2}}{M\left(N_{i}-1\right)}},\;\mathrm{and}$$ (12)

$$R=1-\frac{TEM^{2}}{s^{2}}$$ (13)

where ${\hat{x}}_{ij}$ is the *j*^th^ measurement/estimate of the *i*^th^ participant. *M* is the number of participants and *N*_*i*_ is the number of measurements/estimates for participant *i*. *s* is the standard deviation of the measurements/estimates over the population of the investigated participants.

TEM is the standard deviation of repeated values of an anthropometric quantity, which consists of the variation from the error of the measurement/estimation method and the variation from the investigated population, which is affected by the participants' age, sex and build characteristics. For this reason, the acceptable levels of TEM are hard to determine [25]. However, a previous anthropometric study considered performance to be adequate if the observer's TEM is within ± 2.8 × TEM of the expert's TEM [3]. Here, the photogrammetric method estimates are considered adequate if the TEM of the estimates are within ± 2.8 × TEM of the manual measurements.

*R* refers to the proportion of the variation from the investigated population and ranges from 0 to 1. An *R* value close to one indicates lower variance due to the error of the method and hence higher reliability. In a previous anthropometric study, *R*>0.95 is considered as acceptable performance [26]. This threshold for *R* is likewise used to evaluate the photogrammetric methods in this study.

## Results

### Height

For manual height measurements, the TEM and R were 2.24 mm and 1.00, respectively.

The mean and SD of *MAD*_*i*_ and *MAPD*_*i*_, and, TEM and R for the linear distance estimate of height from each view using camera calibration and using the reference object is presented in Table 2. The optimal view (smallest mean *MAD*_*i*_) is highlighted in bold for both the estimate using camera calibration as well as estimate using the reference object. Using camera calibration, the view that resulted in the smallest mean *MAD*_*i*_ was view 3 (11.78 mm ± 9.37 mm SD). Using the reference object, the view that resulted in the smallest mean *MAD*_*i*_ was view 2 (14.90 mm ± 9.37 mm SD). Finally, using camera calibration, the TEM for all but view 5 and using the reference object, the TEM for view 2 and view 3 were within ± 2.8 × TEM of the manually measured heights indicating adequate performance.

**Table 2:** Performance of height estimation from linear distance from head point to floor line, using camera calibration and reference object.

Views	Camera Calibration						Reference Object					
	*MAD*_*i*_ (mm)		*MAPD*_*i*_ (%)		TEM (mm)	R	*MAD*_*i*_ (mm)		*MAPD*_*i*_ (%)		TEM (mm)	R
	mean	sd	mean	sd			mean	sd	mean	sd		
1	12.17	7.29	0.712	0.420	5.45	0.996	14.98	10.50	0.870	0.589	7.20	0.994
2	12.35	8.31	0.720	0.476	5.65	0.996	**14.90**	**10.01**	0.867	0.568	5.76	0.996
3	**11.78**	**9.37**	0.686	0.530	5.59	0.996	16.55	10.22	0.964	0.583	5.89	0.996
4	12.20	10.65	0.711	0.603	6.21	0.995	20.78	11.61	1.211	0.657	8.27	0.992
5	13.97	12.06	0.813	0.684	7.42	0.993	22.93	12.48	1.335	0.706	7.40	0.994

Bold indicates smallest mean *MAD*_*i*_.

The performance of the estimates of height by the linear regression of linear distance are shown in Table 3 for all combinations of views, using camera calibration as well as using the reference object. The optimal view combination (smallest mean *MAD*_*i*_) is highlighted in bold for both the estimate using camera calibration as well as estimate using the reference object. Using camera calibration with only one view, the smallest mean *MAD*_*i*_ of the linear regression is 3.19 mm smaller (8.59 mm for view 1), than that of the linear distance method (11.78 mm for view 3). Similarly, using the reference object with only one view, improves the smallest mean *MAD*_*i*_ by 3.0 mm (11.90 mm; view 1) compared to the linear distance method (14.90 mm; view 2). The difference in *MAD*_*i*_ between camera calibration and reference object for one view is 3.31 mm; however for the optimal view combinations of more than one view, the difference is approximately 2 mm. In the case of camera calibration, using more than one view does not improve the mean *MAD*_*i*_ of the estimates, however the TEM and R values do improve with more views, and any view combination that includes view 1 has approximately the same performance (mean *MAD*_*i*_ between 8.59 and 8.99 mm across 16 combinations of views). In the case of using the reference object with two views, the mean *MAD*_*i*_ improves by 1.3 mm (10.69 mm ± 7.37 mm SD for view combination {1, 3}) compared to using just one view, and adding more views does not improve the *MAD*_*i*_, but does improve the TEM and R values. Using the reference object, any combination of two or more views that includes view 1 or view 2 has approximately the same performance (mean *MAD*_*i*_ between 10.43 and 10.84 mm, across 24 combinations). Finally, all of the TEM values for camera calibration for view combinations involving view 1 were within ± 2.8 × TEM of the manual measurements, and all R values were > 0.99. Using the reference object, the TEM of all combinations of two or more views (except view combination {4, 5}) were within ± 2.8 × TEM of the manual measurements and all R values were > 0.99.

**Table 3:** Performance of height estimation from linear regression of distance from head point to floor line for all combinations of 1, 2, 3, 4 and 5 views, using camera calibration and floor line.

Views					Camera Calibration						Reference Object					
					*MAD*_*i*_ (mm)		*MAPD*_*i*_ (%)		TEM (mm)	R	*MAD*_*i*_ (mm)		*MAPD*_*i*_ (%)		TEM (mm)	R
					mean	sd	mean	sd			mean	sd	mean	sd		
				1	**8.59**	**6.77**	0.498	0.380	5.31	0.996	10.76	5.81	0.631	0.343	6.56	0.994
				2	9.40	6.89	0.547	0.386	5.55	0.996	**10.60**	**6.43**	0.624	0.386	5.31	0.996
				3	10.41	8.08	0.606	0.454	5.52	0.996	11.05	7.29	0.650	0.435	5.46	0.996
				4	12.57	10.04	0.733	0.569	6.08	0.995	13.02	8.32	0.767	0.486	7.59	0.992
				5	14.49	11.71	0.846	0.663	7.15	0.993	14.18	9.89	0.834	0.575	6.73	0.994
			1	2	**8.69**	**6.99**	0.504	0.392	4.58	0.997	10.66	6.10	0.626	0.364	5.34	0.996
			1	3	8.81	7.19	0.510	0.403	4.61	0.997	10.51	6.52	0.617	0.388	5.14	0.997
			1	4	8.86	7.18	0.513	0.403	4.69	0.997	**10.44**	**6.33**	0.613	0.375	4.80	0.997
			1	5	8.86	7.01	0.513	0.393	5.42	0.996	10.78	6.23	0.632	0.369	6.14	0.995
			2	3	9.45	7.24	0.549	0.406	4.97	0.997	10.51	6.95	0.618	0.415	4.43	0.997
			2	4	9.62	7.15	0.559	0.400	5.42	0.996	10.68	6.70	0.628	0.400	4.72	0.997
			2	5	9.62	6.89	0.559	0.384	5.89	0.995	10.74	6.72	0.632	0.402	5.12	0.997
			3	4	10.61	8.02	0.618	0.449	5.97	0.995	11.18	7.38	0.658	0.439	5.05	0.997
			3	5	10.46	7.73	0.609	0.433	6.64	0.994	11.27	7.34	0.663	0.436	5.77	0.996
			4	5	12.65	9.66	0.737	0.547	7.84	0.992	13.45	8.94	0.792	0.521	7.99	0.992
		1	2	3	**8.83**	**7.23**	0.512	0.405	4.35	0.998	10.62	6.51	0.624	0.388	4.79	0.997
		1	2	4	8.92	7.24	0.517	0.406	4.38	0.997	10.50	6.42	0.617	0.382	4.52	0.997
		1	2	5	8.87	7.08	0.514	0.396	4.67	0.997	10.77	6.36	0.632	0.379	5.25	0.996
		1	3	4	8.95	7.30	0.519	0.409	4.43	0.997	10.48	6.52	0.616	0.387	4.51	0.997
		1	3	5	8.85	7.17	0.512	0.401	4.67	0.997	10.67	6.58	0.626	0.391	5.20	0.996
		1	4	5	8.75	7.00	0.507	0.392	4.96	0.997	**10.43**	**6.25**	0.612	0.370	4.90	0.997
		2	3	4	9.60	7.31	0.558	0.409	5.04	0.997	10.62	6.99	0.625	0.417	4.29	0.998
		2	3	5	9.46	7.13	0.549	0.398	5.16	0.996	10.66	7.02	0.627	0.419	4.52	0.997
		2	4	5	9.56	6.93	0.555	0.387	5.62	0.996	10.84	6.70	0.638	0.400	4.93	0.997
		3	4	5	10.43	7.80	0.607	0.438	6.30	0.995	11.34	7.35	0.667	0.437	5.52	0.996
	1	2	3	4	8.99	7.33	0.521	0.410	4.28	0.998	10.57	6.52	0.621	0.388	4.43	0.997
	1	2	3	5	8.88	7.21	0.514	0.402	4.37	0.997	10.75	6.58	0.632	0.392	4.84	0.997
	1	2	4	5	**8.79**	**7.08**	0.509	0.396	4.62	0.997	10.50	6.34	0.616	0.376	4.55	0.997
	1	3	4	5	8.80	7.15	0.510	0.400	4.62	0.997	**10.45**	**6.45**	0.613	0.382	4.56	0.997
	2	3	4	5	9.45	7.16	0.549	0.400	5.13	0.997	10.75	6.99	0.633	0.417	4.46	0.997
1	2	3	4	5	**8.84**	**7.19**	0.512	0.402	4.46	0.997	**10.55**	**6.48**	0.619	0.385	4.41	0.997

Bold indicates smallest mean *MAD*_*i*_.

The performance of the estimates of height by the linear regression of bounding box height are shown in Table 4 for all combinations of views, using camera calibration as well as using the reference object. Using camera calibration with only one view, the mean *MAD*_*i*_ of the linear regression is comparable (11.90 mm for view 1), to the linear distance method (11.78 mm for view 3). Using the reference object method with only one view, improves the mean *MAD*_*i*_ by 3.03 mm (11.87 mm; view 1) compared to the linear distance method (14.90 mm; view 2). The difference in *MAD*_*i*_ between camera calibration and reference object for one view is only 0.03 mm with the reference object out-performing camera calibration, however for the optimal combinations (combinations with smallest mean *MAD*_*i*_) of more than one view, the difference in *MAD*_*i*_ is between 0.34 and 0.65 mm with the camera calibration out-performing the reference object. In the case of camera calibration, using two views improves the mean *MAD*_*i*_ of the estimates, however adding further views does not improve the mean *MAD*_*i*_, while the TEM and R improve for combinations of more views. Any combination of two or more views that includes view 1 has approximately the same performance (mean *MAD*_*i*_ between 10.69 and 11.07 mm across 15 combinations of views), with the best performance being for the combination of view 1 and 3 (mean *MAD*_*i*_ 10.69 ± 7.37 mm SD). In the case of using the reference object, using two views instead of just one view, the mean *MAD*_*i*_ improves by 0.84 mm (11.03 mm ± 6.28 mm SD for combination of views 1 and 3), however adding more views does not improve the *MAD*_*i*_, but does improve the TEM and R values. Using the reference object, any combination of two or more views that includes view 1 has approximately the same performance (mean *MAD*_*i*_ between 11.19 and 11.97 mm, across 16 combinations). The TEM values for camera calibration or reference object with combinations of 3 or more views (except view combinations {1, 3, 4, 5} and {2, 3, 4, 5}) were within ± 2.8 × TEM of the manual measurements, and all R values were > 0.98.

**Table 4:** Performance of height estimation from linear regression of bounding box height for all combinations of 1, 2, 3, 4 and 5 views, using camera calibration and reference object.

Views					Camera Calibration						Reference Object					
					*MAD*_*i*_ (mm)		*MAPD*_*i*_ (%)		TEM (mm)	R	*MAD*_*i*_ (mm)		*MAPD*_*i*_ (%)		TEM (mm)	R
					mean	sd	mean	sd			mean	sd	mean	sd		
				1	**11.90**	**7.49**	0.692	0.421	9.03	0.989	**11.87**	**6.78**	0.693	0.389	9.01	0.989
				2	12.65	7.14	0.737	0.402	7.68	0.992	12.80	7.17	0.753	0.424	7.45	0.993
				3	15.13	9.65	0.884	0.553	6.57	0.994	13.97	10.22	0.823	0.607	6.50	0.994
				4	18.75	12.22	1.098	0.710	7.56	0.992	17.29	11.24	1.017	0.666	7.58	0.992
				5	19.59	14.06	1.148	0.805	8.15	0.991	18.77	11.72	1.104	0.674	8.53	0.990
			1	2	11.07	7.27	0.643	0.407	6.47	0.994	11.97	6.10	0.701	0.353	6.49	0.994
			1	3	**10.69**	**7.37**	0.620	0.411	5.76	0.996	11.53	6.43	0.676	0.373	5.88	0.995
			1	4	10.72	7.55	0.623	0.425	6.13	0.995	**11.03**	**6.28**	0.647	0.364	6.03	0.995
			1	5	10.77	8.03	0.625	0.453	6.82	0.994	11.09	6.32	0.648	0.363	6.78	0.994
			2	3	12.87	7.58	0.751	0.427	6.26	0.995	12.73	7.99	0.750	0.474	5.96	0.995
			2	4	12.92	7.38	0.753	0.416	6.77	0.994	12.67	7.70	0.746	0.456	6.24	0.995
			2	5	12.83	7.59	0.748	0.425	6.74	0.994	12.46	7.60	0.733	0.447	6.27	0.995
			3	4	15.26	9.64	0.892	0.552	6.81	0.994	14.14	10.26	0.833	0.609	6.06	0.995
			3	5	15.53	10.00	0.907	0.572	6.35	0.995	14.33	10.26	0.844	0.605	6.03	0.995
			4	5	18.96	12.86	1.110	0.743	6.83	0.994	17.61	11.69	1.035	0.687	6.81	0.994
		1	2	3	10.81	7.37	0.628	0.410	5.31	0.996	11.68	6.50	0.685	0.378	5.44	0.996
		1	2	4	10.83	7.52	0.629	0.422	5.44	0.996	11.32	6.37	0.664	0.371	5.45	0.996
		1	2	5	**10.69**	**7.93**	0.620	0.446	5.53	0.996	11.30	6.37	0.662	0.368	5.59	0.996
		1	3	4	10.79	7.56	0.627	0.423	5.36	0.996	11.33	6.45	0.665	0.375	5.40	0.996
		1	3	5	10.70	7.83	0.620	0.438	5.46	0.996	11.29	6.51	0.662	0.377	5.55	0.996
		1	4	5	10.77	7.85	0.625	0.442	5.95	0.995	**11.19**	**6.41**	0.655	0.370	5.88	0.995
		2	3	4	13.06	7.64	0.762	0.430	6.14	0.995	12.75	8.00	0.751	0.474	5.79	0.996
		2	3	5	13.06	7.72	0.761	0.433	6.17	0.995	12.67	7.82	0.746	0.460	5.94	0.995
		2	4	5	12.77	7.53	0.744	0.421	6.83	0.994	12.54	7.53	0.738	0.442	6.38	0.995
		3	4	5	15.09	9.95	0.881	0.564	7.23	0.993	14.34	10.10	0.844	0.594	6.46	0.994
	1	2	3	4	10.92	7.54	0.634	0.422	5.09	0.997	11.47	6.51	0.673	0.379	5.19	0.996
	1	2	3	5	10.80	7.81	0.626	0.437	5.03	0.997	11.42	6.55	0.670	0.379	5.16	0.996
	1	2	4	5	**10.77**	**7.90**	0.625	0.444	5.26	0.996	11.39	6.45	0.668	0.373	5.29	0.996
	1	3	4	5	10.77	7.90	0.624	0.442	5.27	0.996	**11.38**	**6.57**	0.667	0.380	5.32	0.996
	2	3	4	5	12.86	7.75	0.749	0.432	6.33	0.995	12.67	7.74	0.746	0.455	6.05	0.995
1	2	3	4	5	**10.86**	**7.90**	0.630	0.442	4.98	0.997	**11.51**	**6.60**	0.675	0.382	5.08	0.997

Bold indicates smallest mean *MAD*_*i*_.

### MUAC

The TEM and R for the manual MUAC measurements were 3.76 mm and 0.99, respectively.

The performance of the three shape models for estimating MUAC, for all views in the case of the circle, and for all combinations of two views in the case of the ellipse and the combination of the ellipse and rectangle, are shown in Table 5. The optimal view combination (smallest mean *MAD*_*i*_) is highlighted in bold for both the estimate using camera calibration as well as estimate using the reference object. Using the camera calibration, the ellipse method was found to be the most accurate of the three shape models with a mean *MAD*_*i*_ of 9.35 mm ± 5.07 mm SD (optimal view combination {1, 3}), compared to 12.51 mm ± 9.66 mm SD for the circle model (optimal view 4) and 12.81 mm ± 8.60 mm SD for the combined rectangle and ellipse model (optimal view combination {1, 4}). The same view combinations had the smallest *MAD*_*i*_ when using the reference object and the mean *MAD*_*i*_ was approximately 1.5–2.5 mm greater than in the case of using camera calibration. Finally, the TEM values for almost all shape models and view combinations were within ± 2.8 × TEM of the manual measurements, and almost all R values were greater than 0.95.

**Table 5:** Performance of MUAC estimation from shape models using camera calibration and reference object.

Shape	Views		Camera Calibration						Reference Object					
			*MAD*_*i*_ (mm)		*MAPD*_*i*_ (%)		TEM (mm)	R	*MAD*_*i*_ (mm)		*MAPD*_*i*_ (%)		TEM (mm)	R
			mean	sd	mean	sd			mean	sd	mean	sd		
Circle		1	51.99	24.73	17.47	7.31	10.72	0.894	51.16	26.77	17.18	8.05	11.32	0.893
		2	42.77	18.52	14.29	4.87	6.22	0.986	42.61	19.94	14.24	5.49	7.73	0.979
		3	60.78	18.93	20.44	4.56	4.17	0.994	60.07	19.60	20.22	5.01	6.23	0.987
		4	**12.51**	**9.66**	4.19	2.98	6.59	0.967	**14.18**	**10.31**	4.76	3.21	7.34	0.960
		5	76.96	48.83	25.23	14.66	10.44	0.914	77.07	48.61	25.26	14.52	10.70	0.907
Ellipse	1	2	11.82	7.11	3.96	2.27	6.00	0.979	13.52	9.79	4.53	3.15	7.19	0.971
	1	3	**9.35**	**5.07**	3.19	1.67	4.98	0.985	**11.28**	**7.86**	3.85	2.61	6.35	0.976
	1	4	24.63	12.47	8.17	3.52	5.79	0.964	24.83	14.40	8.23	4.23	6.65	0.955
	1	5	63.50	28.11	21.03	7.52	7.28	0.886	63.11	28.50	20.89	7.63	7.83	0.874
	2	3	51.89	16.46	17.41	3.73	3.48	0.996	51.43	17.68	17.26	4.41	5.79	0.988
	2	4	22.38	7.86	7.58	2.27	3.69	0.993	22.30	10.56	7.57	3.31	5.40	0.984
	2	5	17.21	12.90	5.76	4.20	5.03	0.977	17.86	12.44	5.95	3.95	6.06	0.967
	3	4	31.65	12.54	10.75	3.73	4.36	0.991	31.25	14.02	10.63	4.36	5.84	0.983
	3	5	15.86	10.51	5.44	3.50	5.10	0.980	15.44	11.11	5.28	3.62	6.13	0.970
	4	5	36.82	26.36	12.01	7.85	6.30	0.950	37.12	26.29	12.10	7.77	6.82	0.941
Ellipse/	1	2	35.85	16.53	12.22	5.14	7.01	0.977	36.94	18.32	12.61	5.73	8.32	0.969
Rectangle	1	3	46.45	10.86	15.84	2.98	5.97	0.983	46.48	14.76	15.86	4.53	7.46	0.974
	1	4	**13.81**	**8.60**	4.94	3.27	6.70	0.962	**16.22**	**10.42**	5.72	3.81	7.68	0.954
	1	5	36.00	24.00	11.72	6.89	8.45	0.883	35.67	24.42	11.59	7.01	9.08	0.871
	2	3	98.89	23.36	33.42	4.24	3.97	0.996	98.37	24.40	33.26	5.01	6.59	0.988
	2	4	65.10	12.48	22.18	2.58	4.20	0.993	64.94	14.82	22.14	3.81	6.14	0.984
	2	5	25.68	14.77	9.14	5.74	6.07	0.974	25.16	15.54	8.96	5.92	7.17	0.963
	3	4	75.47	17.09	25.72	4.26	5.01	0.990	75.01	18.41	25.58	4.99	6.66	0.983
	3	5	34.87	19.10	12.28	7.08	6.27	0.975	34.86	17.97	12.26	6.71	7.34	0.966
	4	5	23.61	16.85	8.08	5.50	7.38	0.947	23.73	16.98	8.09	5.48	7.96	0.939

Bold indicates smallest mean *MAD*_*i*_.

The results for the linear regression MUAC estimate using both the camera calibration and the reference object are presented in Table 6 for all combinations of views. A similar trend is observed here as was seen in the performance of linear regression for height estimation. Even with only one view, the linear regression MUAC estimate outperforms all of the shape models (mean *MAD*_*i*_ of only 6.47 mm ± 4.86 mm SD for view 3 using camera calibration, and mean *MAD*_*i*_ 7.55 mm ± 5.49 mm SD using the reference object). Adding a second view to the linear regression improves the mean *MAD*_*i*_ by 1.85 mm using the camera calibration (4.62 mm ± 3.25 mm SD for optimal view combination {1, 3}), and adding another one, two or three views only improves the performance by, at most, another 0.48 mm (4.14 mm ± 2.73 mm SD, optimal view combination {1, 2, 3, 4}). Similarly, using the reference object, adding a second view improves the mean *MAD*_*i*_ by 0.93 mm (6.62 mm ± 4.64 mm SD, optimal view combination {2,3}); however adding more views does not improve the mean *MAD*_*i*_. Using the reference object, any combination of one or more views that includes view 3 has approximately the same performance (mean *MAD*_*i*_ ranging from 6.62 mm– 7.62 mm, across 16 combinations). Finally, the TEM values for almost all view combinations was within ± 2.8 × TEM of the manual measurements, and almost all R values were greater than 0.95.

**Table 6:** Performance of MUAC estimation from linear regression of arm width for combinations of 1, 2, 3, 4 and 5 views using camera calibration and reference object.

Views					Camera Calibration						Reference Object					
					*MAD*_*i*_ (mm)		*MAPD*_*i*_ (%)		TEM (mm)	R	*MAD*_*i*_ (mm)		*MAPD*_*i*_ (%)		TEM (mm)	R
					mean	sd	mean	sd			mean	sd	mean	sd		
				1	21.57	13.98	7.43	4.65	9.12	0.893	22.84	14.63	7.86	4.85	8.76	0.891
				2	8.14	5.33	2.76	1.72	4.30	0.986	9.86	5.79	3.34	1.87	5.22	0.979
				3	**6.47**	**4.86**	2.20	1.63	2.83	0.994	**7.55**	**5.49**	2.58	1.86	4.20	0.987
				4	12.52	10.18	4.21	3.11	6.19	0.967	14.22	10.56	4.79	3.27	6.66	0.960
				5	31.41	22.63	10.89	8.11	1.16	0.913	31.42	22.65	10.89	8.11	1.18	0.908
			1	2	8.20	5.37	2.78	1.74	4.35	0.986	9.61	5.80	3.26	1.93	5.59	0.976
			1	3	**4.62**	**3.25**	1.59	1.14	3.21	0.993	7.24	4.79	2.47	1.62	4.29	0.986
			1	4	9.22	6.32	3.14	2.08	6.26	0.970	12.35	8.30	4.17	2.66	6.72	0.963
			1	5	20.52	13.07	7.07	4.46	10.12	0.881	21.86	13.99	7.51	4.68	9.86	0.875
			2	3	4.72	3.81	1.61	1.33	2.35	0.996	**6.62**	**4.64**	2.26	1.60	3.94	0.989
			2	4	4.93	3.31	1.69	1.15	3.18	0.993	7.12	5.56	2.42	1.87	4.55	0.985
			2	5	8.04	5.24	2.73	1.73	4.23	0.987	9.65	5.81	3.26	1.89	5.26	0.979
			3	4	6.58	5.04	2.24	1.70	2.86	0.994	7.52	5.94	2.58	2.04	4.28	0.986
			3	5	6.34	4.68	2.16	1.60	2.85	0.994	7.62	5.41	2.62	1.85	4.15	0.987
			4	5	12.58	9.08	4.23	2.76	6.33	0.967	14.12	10.11	4.77	3.18	6.69	0.961
		1	2	3	**4.28**	**3.11**	1.47	1.09	2.51	0.996	6.74	4.64	2.30	1.60	3.92	0.989
		1	2	4	4.78	3.17	1.64	1.09	3.13	0.993	7.15	5.62	2.43	1.90	4.63	0.984
		1	2	5	8.13	5.29	2.75	1.74	4.19	0.987	9.58	5.80	3.25	1.92	5.48	0.977
		1	3	4	4.70	3.27	1.61	1.14	3.25	0.992	7.37	5.14	2.52	1.75	4.31	0.986
		1	3	5	4.69	3.32	1.61	1.16	3.22	0.993	7.35	4.91	2.51	1.66	4.28	0.986
		1	4	5	9.32	6.38	3.17	2.09	6.21	0.970	12.45	8.40	4.21	2.69	6.65	0.963
		2	3	4	4.48	3.43	1.53	1.19	2.43	0.996	6.75	4.94	2.31	1.70	3.93	0.989
		2	3	5	4.75	3.84	1.62	1.34	2.35	0.996	**6.73**	**4.75**	2.30	1.64	3.94	0.989
		2	4	5	4.93	3.34	1.69	1.16	3.21	0.993	7.22	5.65	2.45	1.90	4.56	0.985
		3	4	5	6.50	4.85	2.21	1.64	2.85	0.994	7.61	5.88	2.62	2.03	4.21	0.987
	1	2	3	4	**4.14**	**2.73**	1.42	0.96	2.44	0.996	6.87	4.97	2.34	1.71	3.91	0.989
	1	2	3	5	4.36	3.15	1.50	1.10	2.53	0.995	**6.84**	**4.75**	2.34	1.64	3.94	0.989
	1	2	4	5	4.85	3.19	1.67	1.10	3.14	0.993	7.22	5.71	2.45	1.94	4.65	0.984
	1	3	4	5	4.76	3.35	1.63	1.16	3.24	0.993	7.48	5.23	2.56	1.78	4.31	0.986
	2	3	4	5	4.49	3.38	1.53	1.18	2.44	0.996	6.86	5.03	2.34	1.73	3.93	0.989
1	2	3	4	5	**4.21**	**2.75**	1.44	0.97	2.43	0.996	**6.98**	**5.06**	2.38	1.74	3.92	0.989

Bold indicates smallest mean *MAD*_*i*_

## Discussion

In this work, it has been shown that linear regression of silhouette height measurements from photos taken from different views with a calibrated camera can be used to estimate a person's height accurately. This approach has been shown to be more accurate than measuring a linear distance from the top of the participant’s head to the floor using a single photo, but not more accurate than linear regression of the linear distance using multiple views. In general, for both linear regression methods of height estimation, combinations of views involving the frontal view (view 1) gave the smallest mean *MAD*_*i*_’s (approximately 8–10 mm using camera calibration) and TEM and R values were within adequate limits according to [3].

It has also been shown that the linear regression of mid-upper arm radius measurements from photos taken from different views can be used to estimate MUAC accurately. This approach has been shown to be superior to shape model methods which have previously been reported in the literature for other circumferential measurements. In general, for the linear regression method of MUAC estimation, combinations of views involving the side view (view 3) gave the smallest mean *MAD*_*i*_’s (approximately 4–6 mm using camera calibration) and TEM and R values were within adequate limits according to [3].

It was also observed that in general, when adding views to the linear regression models, the largest improvement in mean *MAD*_*i*_ for the optimal view combinations was observed when the number of views used increased from one to two, and that the TEM and R improved when more views were used. Finally, *MAD*_*i*_ when using the reference object is a maximum of 2 to 3 mm larger than the *MAD*_*i*_ when using camera calibration.

### Big picture

Anthropometric measurements are important for indicating general health and wellbeing. Height and MUAC are two common indicators of health and nutrition status in children and adults [3–5]. Typically, these values are measured manually by trained observers, which is time consuming and can result in unreliable measurements. Furthermore, in developing countries where resources are limited, the MUAC is the only measure used for malnutrition assessment of children [27,28], despite several measurements being recommended by the WHO [6]. The work here has shown that it is possible to extract height and MUAC accurately from photos taken from different views. With some further work, such a technique has the potential to be used for malnutrition assessment, and could reduce the measurement time and the required expertise of the observer–it may also be possible to implement such a method on a mobile device. All these things contribute to greater access to malnutrition screening and therefore higher chance of treatment which could have a large impact locally in developing countries and ultimately globally as well.

### Practical considerations

#### Cost and benefit of additional views

The general trend for the linear regression estimates of height and MUAC indicates that estimates using more photos (from more views) are more accurate. However, the benefit gained decreases as the number of photos increases. It is important also to consider that the more photos required, the greater the time for measurement and also the more complex the photo capture procedure. Furthermore, photos from some view angles are more difficult to capture than others, for example, it might be difficult to capture photos where the subject is at 45° with respect to the camera (as in views 2 and 4), as compared to capturing a photo from the frontal or side view (as in views 1 and 3 respectively). Misalignment errors, which are more likely to be introduced in the cases of views 2 and 4, may be a contributing factor in larger MAD for height and MUAC estimates which use these views–for height, generally smaller error was observed for view combinations that include view 1, and for MUAC, generally smaller error was observed for view combinations that include view 3.

For these reasons, the combination of view 1 and view 3 (which correspond to the frontal and side views of the participants) is considered here to be the best combination for height and MUAC estimation using linear regression methods. Using camera calibration, the combination of view 1 and view 3 gave mean *MAD*_*i*_ of 10.69 mm ± 7.37 mm SD and 4.62 mm ± 3.25 mm SD for height from linear regression of the bounding box and MUAC from linear regression of mid-upper arm width, respectively, and was considered to have adequate accuracy based on TEM and R values. Also, using the reference object, the combination of view 1 and view 3 gave mean *MAD*_*i*_ of 11.53 mm ± 6.43 mm SD and 7.24 mm ± 5.49 mm SD for height from linear regression of the bounding box and MUAC from linear regression of mid-upper arm width, respectively, and was considered to have adequate accuracy based on TEM and R values.

#### Calibrated camera vs. reference object

In general, two methods exist for spatial measurement from photographs: (i) the calibrated camera method and (ii) the reference object method. Camera calibration is the process of estimating the camera intrinsic parameters, distortion coefficients and extrinsic parameters. This is typically done by taking numerous photos of a special calibration pattern in different locations and orientations, then applying known algorithms. The estimated parameters are unique to each camera. In contrast, the reference object method only requires that a reference object of known proportions be placed in the photograph in the same plane as the object to be measured—this is less time consuming than the calibrated camera method and more novice-friendly.

The calibrated camera method is reported in the literature as being superior to the reference object method with respect to spatial measurement accuracy. This was also found to be the case in this work; however, the error was only approximately 2 to 3 mm larger when using the reference object method. The benefit of greater estimation accuracy using the camera calibration, is at odds with the reduced setup complexity (and observer expertise) of using the reference object. It is recommended that the decision of whether to use camera calibration or the reference object be dependent on the application–for applications requiring higher accuracy with a trained observer using a single camera for anthropometric measurements of numerous subjects with a fixed setup, the camera calibration method is recommended; alternatively, for applications where there are multiple naive observers using different cameras and where portability may be required, the reference object method is recommended.

#### Camera quality

The camera used to collect the photo data was a Canon IXUS 110 IS digital camera (auto focus, auto exposure time, ISO-200, zero zoom, no image stabilization) with a resolution of 4000×3000 pixels. The camera specifications and settings, and the environmental conditions (e.g. changing lighting conditions between participants) are by no means ideal. Photo capture under ideal conditions however was not the goal of this study and the introduction of variance into the photo capture process serves to prove the robustness of the proposed linear regression technique for height and MUAC estimation and highlight the potential for applying the technique in the real-world where conditions are unlikely to be ideal. There is no doubt that a more controlled environment (e.g. using image stabilization, fixed lighting conditions) and a higher resolution camera with a more sophisticated lens and sensor would improve estimation accuracy–however, it is again recommended that such specifications be determined depending on the application.

### Comparison to previous literature

Photogrammetric height estimation has been reported in the literature using a variety of methods. Height has been estimated using single photos containing a reference object and making use of the vanishing line, however only a single example of height measurement was reported [29]. A single uncalibrated photo was used [30], in addition to probabilistic and statistical knowledge of human anthropometry in a Bayesian-like framework; however, the method had only slightly better accuracy than by assuming that all subjects had a height equal to the population mean. Another method used a single photo (frontal) and a reference ruler backdrop to measure segment lengths and height [14], and the reported mean difference against manual measurement for height across 21 subjects was 8 mm ± 5 mm. The smallest mean MAD in the current work is 8.59 mm ± 6.77 mm SD (linear regression of linear distance with camera calibration using view 1, Table 3), which is comparable to [14]. In the case of linear regression of the bounding box, the smallest mean MAD was 10.69 ± 7.37 mm SD (camera calibration using view combination {1, 3}, Table 4). The larger error in the linear regression of the bounding box is most likely due to not identifying the location of the floor, whereas for the linear regression of the linear distance the position of the floor is known with some degree of accuracy, and in [14], the floor at the center of the feet was marked. While the error is larger for the linear regression of the bounding box (only 2.1 mm larger mean MAD than linear regression of linear distance from head to floor for calibrated camera, and only 0.6 mm larger mean MAD for reference object), this approach does allow greater flexibility in the location of the checkerboard/reference object, and in the case of using a reference object, allows greater flexibility in the choice of reference object shape.

Body circumference measurements have been estimated using shape models from photos from two (front and side [2,16]) or three (front, side and back [17]) views. The smallest MAD reported was using the ellipse shape model to estimate arm circumference (under the pit), which was 15.78 mm [16]. To the best of the authors' knowledge, only one attempt to estimate MUAC has been made [20], but there have been no reports on the accuracy of the estimate. In this work, the smallest mean *MAD*_*i*_ for the MUAC estimate using a shape model was 9.35 mm ± 5.07 mm SD (ellipse shape model applied to views 1 and 3 using camera calibration; Table 5). The linear regression model outperformed the ellipse shape model even when using only one photo (mean *MAD*_*i*_ 6.47 mm ± 4.86 mm SD for view 3 using camera calibration; mean *MAD*_*i*_ 7.55 mm ± 5.49 mm SD for view 3 using the reference object; Table 6), and when using two photos as in the ellipse shape model, the mean *MAD*_*i*_ was just 4.62 mm ± 3.25 mm SD (for the combination of view 1 and 3 using camera calibration; Table 6).

More recent work in the area of photogrammetry of the human body has focused on using multiple cameras [31,32], depth cameras [33,34], or 3D scanners [35], with the general aim being to reconstruct a 3D model of the participant, but with few extracting specific anthropometric measurements. Setups with multiple cameras are cumbersome and not likely to be easily portable, which limits their use and 3D model reconstruction and curve fitting is computationally expensive. Furthermore, while the availability of devices incorporating depth cameras, and 3D scanners are becoming more available, they are still considered specialist devices and the technology is yet to be incorporated into a large range of mobile devices. Furthermore, the penetration of new technology in developing countries is a number of years behind that in developed countries—for example projections of smart-phone use in emerging markets is predicted to 50% penetration in Africa [13] and the Middle-East by 2020 [12] and 75% penetration by the year 2024 [12]. Therefore developing anthropometric measurement techniques that rely on relatively new technologies may not be feasible for applications in developing countries where a secondary goal must be to improve accessibility and reduce cost.

### Future work

The scope of the work here was to show that linear regression of distance measurements from photographs taken from multiple views was suitable anthropometric estimation of height and MUAC. However, for the proposed method to be practically useful, particularly in a resource-constrained environment (e.g. for malnutrition assessment in developing countries), a number of improvements are required. The most important future work is the automation of landmark detection (reference object, bounding box or top of head and floor, and edges of the mid-upper arm). In the current work, these were manually labeled, however to reduce complexity (and improve usability) and also to minimize user-input and time required to perform the measurement, automation of this processing step is essential. Secondly, the data set here consisted of photographs of healthy adults (none were considered to be undernourished by clinical standards). While there are many possible applications for photogrammetric anthropometry, in the case of malnutrition assessment, the proposed method will need to be validated on photographs of undernourished adults and further validated on photographs of (malnourished) children, to determine the suitability of the method as an alternative to manual measurement in developing countries.

### Conclusions

Anthropometric measurements are important for assessing human health. In this study, two such measurements, namely height and MUAC, have been estimated by applying linear regression to distance measurements made from manually selected landmarks in photos of adults taken from multiple view angles. The distances were computed in real world units using camera calibration as well as a reference object. The linear regression estimates outperformed estimates from other photo-based methods previously reported in the literature for height and body circumferences. The optimal combination of views for the linear regression was found to be the combination of frontal and side views, considering both the estimate accuracy and the number of photos required. Using these views, the linear regression estimates of height and MUAC (whether using camera calibration or a reference object) were within the accuracy required for nutrition assessment. It was also found that approaches that simplify the requirements for capturing photos and therefore reduce the time required for capturing photos and the expertise required of the observer (i.e. using a reference object rather than camera calibration, and eliminating the need to identify the location of the floor for height estimation) add only 2–3 mm of error to the estimates. Future work will focus on automating the landmark detection, and validating the methods on populations that include undernourished adults and children of all nutrition statuses. These future works will improve the practicality of this method as a potential tool for nutrition assessment for use by novice users.
